# Supra­molecular hydrogen-bonding patterns in a 1:1 co-crystal of the N(7)—H tautomeric form of *N* ^6^-benzoyl­adenine with 4-hy­droxy­benzoic acid

**DOI:** 10.1107/S2056989017002171

**Published:** 2017-02-17

**Authors:** Robert Swinton Darious, Packianathan Thomas Muthiah, Franc Perdih

**Affiliations:** a School of Chemistry, Bharathidasan University, Tiruchirappalli 620 024, Tamilnadu, India; b Faculty of Chemistry and Chemical Technology, University of Ljubljana, Večna pot 113, PO Box 537, SI-1000 Ljubljana, Slovenia

**Keywords:** crystal structure, hydrogen bond, dihedral angle, coplanar, supra­molecular inter­action

## Abstract

The asymmetric unit of the title co-crystal, C_12_H_9_N_5_O·C_7_H_6_O_3_, contains one mol­ecule of *N* ^6^-benzoyl­adenine (BA) and one mol­ecule of 4-hy­droxy­benzoic acid (HBA). The *N* ^6^-benzoyl­adenine (BA) has an N(7)—H tautomeric form with nonprotonated N-1 and N-3 atoms. This tautomeric form is stabilized by a typical intra­molecular N—H⋯O hydrogen bond between the carbonyl (C=O) group and the N(7)—H hydrogen on the Hoogsteen face of the purine ring, forming a graph-set *S*(7) ring motif. The primary robust *R*_2_^2^(8) ring motif is formed in the Watson–Crick face *via* N—H⋯O and O—H⋯N hydrogen bonds (involving N1, N6—H and the carboxyl group of HBA). Weak inter­actions, such as, C—H⋯π and π–π are also observed in this crystal structure.

## Abstract 2

The asymmetric unit of the title co-crystal contains one mol­ecule of *N* ^6^-benzoyl­adenine (BA) and one mol­ecule of 4-hy­droxy­benzoic acid (HBA). The *N* ^6^-benzoyl­adenine (BA) has an N(7)—H tautomeric form with non-protonated N-1 and N-3 atoms. This tautomeric form is stabilized by a typical intra­molecular N—H⋯O hydrogen bond on the Hoogsteen face of the purine ring. The primary robust 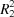
(8) ring motif is formed in the Watson–Crick face *via* N—H⋯O and O—H⋯N hydrogen bonds.

## Chemical context   

Adenine is one of the major nucleobases and some of its *N* ^6^-derivatives have plant hormone (kinetin) (Tr). They also offer a variety of hydrogen-bonding donor and acceptor sites (McHugh & Erxleben, 2011 ▸; Imaz *et al.*, 2011 ▸). 4-Hy­droxy­benzoic acid is also a promising hydrogen-bond donor with the ability to form co-crystals with other organic mol­ecules (Vishweshwar *et al.*, 2003 ▸). It is used as an anti­microbial paraben (Barker & Frost, 2001 ▸). The present study investigates co-crystal formation between *N* ^6^- benzoyl­adenine and 4-hy­droxy­benzoic acid.
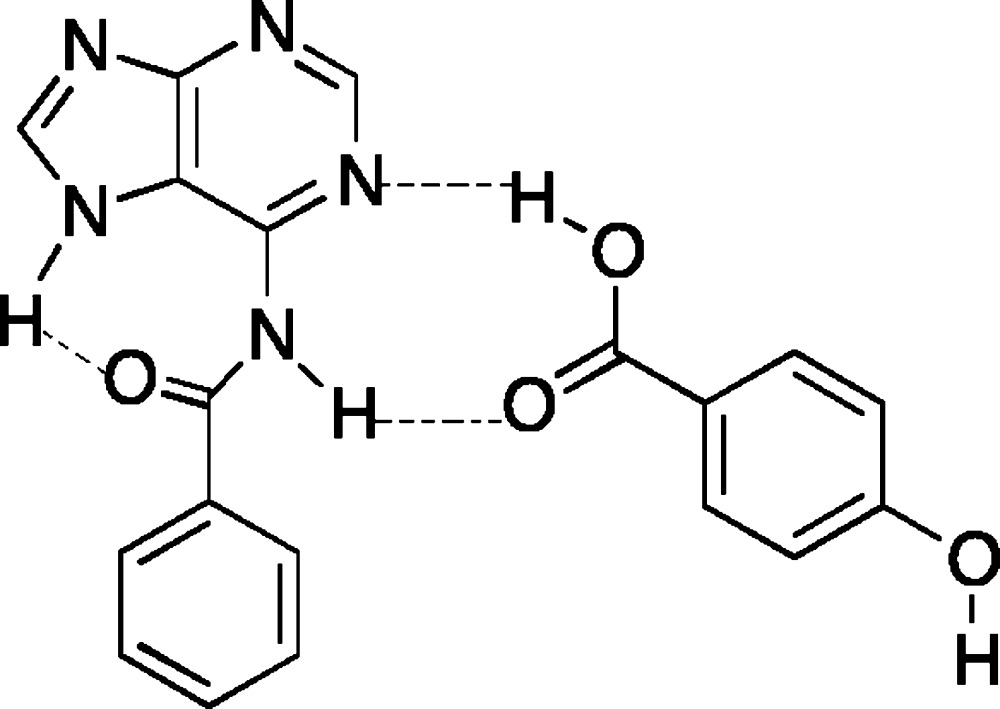


## Structural commentary   

**Figure 1:**
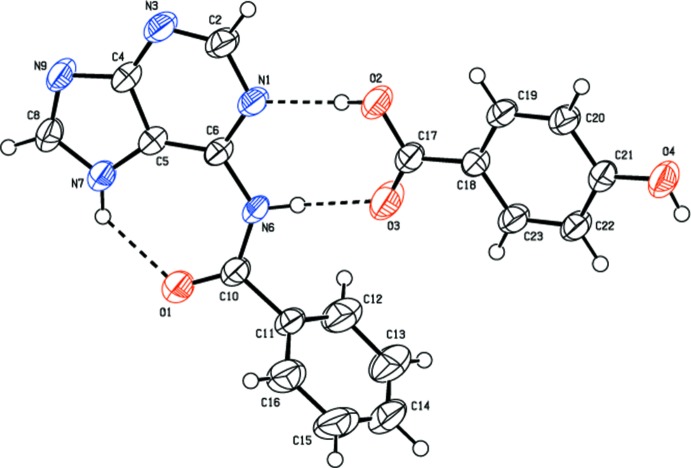
The asymmetric unit of the title compound, showing the atom-numbering scheme. Displacement ellipsoids are drawn at the 50% probability level. Dashed lines represent hydrogen bonds.

In the title co-crystal (I), the asymmetric unit contains one *N* ^6^-benzoyl­adenine (BA) mol­ecule and one 4-hy­droxy­benzoic acid (HBA) mol­ecule (Fig. 1 ▸). The bond angle at N7 [C8—N7—C5 = 106.93 (17)°] is wider than at N9 [C8—N9—C4 = 104.19 (16)°]. In addition, the C8—N7 bond [1.343 (2)Å] is longer than C8—N9 [1.319 (3) Å]. These values agree with those reported earlier for the crystal structure of *N* ^6^-benzoyl­adenine (Raghunathan & Pattabhi, 1981 ▸). In the title co-crystal, the *N* ^6^-benzoyl­adenine also exists in the N(7)—H tautomeric form with non-protonated N1, N3 and N9 atoms. In the crystal structures of *N* ^6^-benzoyl­adenine (Raghunathan & Pattabhi, 1981 ▸), *N* ^6^-benzoyl­adenine-3-hy­droxy­pyridinium-2-carboxyl­ate (1:1) and *N* ^6^-benzoyl adenine-dl-tartaric acid (1:1) (Karthikeyan *et al.*, 2015 ▸), *N* ^6^-benzoyl­adeninium nitrate (1:1) (Karthikeyan *et al.*, 2016 ▸), *N* ^6^-benzoyl adenine–adipic acid (1:0.5) (Swinton Darious *et al.*, 2016 ▸) and the title compound (I), the *N* ^6^-substituent is distal to the N1 and *syn* to the adenine nitro­gen atom N7. This may be due to the participation of the N7 atom in N7—H7⋯O1*A* intra­molecular hydrogen bond (Table 1 ▸) with an *S*(7) ring motif in the Hoogsteen face. In contrast, it may be noted that in the crystal structure of *N* ^6^-benzyl­adenine, (where no intra­molecular hydrogen bond is present) the *N* ^6^-substituent is *syn* to N1 and distal to N7 and the adenine moiety exists in the N(9)—H tautomeric form (Raghunathan *et al.*, 1983 ▸). The dihedral angle between the benzene ring and the carboxyl group of HBA is 1.5 (3)°, indicating that the benzene ring and the carboxyl group are nearly coplanar. A comparison of dihedral angles and the C6—N6—C10—C11 torsion angle reported for various *N* ^6^-benzoyl­adenine-containing crystal structures is given in Table 2 ▸.

## Supra­molecular features   

**Figure 2:**
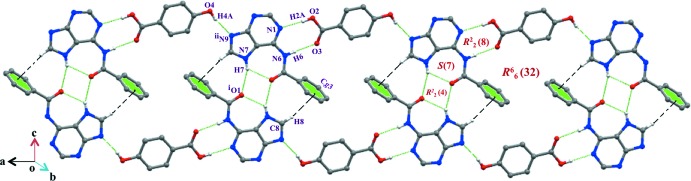
The formation of a supra­molecular three-dimensional large ring structure in the title compound.

**Figure 3:**
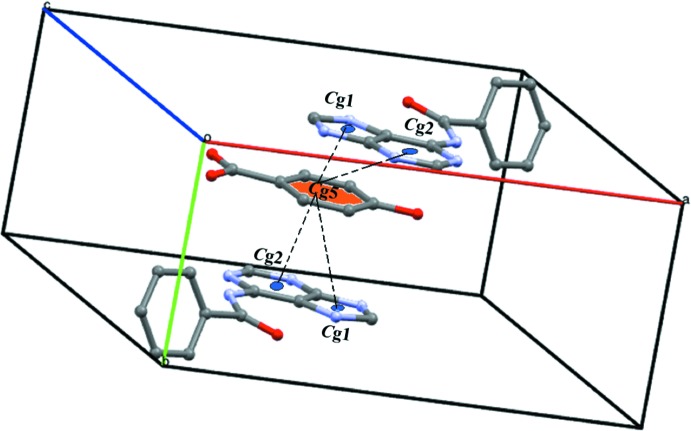
A view of the homo/hetero-stacking inter­actions in the title compound.

The robust 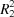
(8) ring motif is formed in the Watson–Crick face (N1 and N6 atoms) *via* N—H⋯O and O—H⋯N hydrogen bonds involving the carboxyl group of HBA. The N7 atom is a bifurcated donor and the carbonyl oxygen atom acts as a double acceptor for the N—H⋯O hydrogen bonds. Inversion-related BA mol­ecules form dimers through an array of hydrogen bonds, generating ring motifs, and these dimers are doubly bridged by inversion-related HBA mol­ecules (Fig. 2 ▸). A large *R*_6_^6^(32) supra­molecular ring is formed along the *c*-axis direction. A weak C8—H8⋯π inter­action is also present. Further consolidation of the structure is provided by homo and hetero π–π stacking inter­actions [*Cg*1⋯*Cg*5(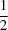 − *x*, 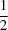 + *y*, 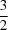 − *z*) = 3.5580 (13) Å, *Cg*2⋯*Cg*5(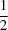 − *x*, −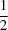 + *y*, 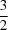 − *z*) = 3.6508 (12) Å; *Cg*1, *Cg*2 and *Cg*5 are the centroids of the imidazole ring, the pyrimidine ring and the benzene ring of HBA, respectively] (Fig. 3 ▸).

## Database survey   

The neutral mol­ecule *N* ^6^-benzoyl­adenine was first reported by Raghunathan & Pattabhi (1981 ▸). Various salts and co-crystals of *N* ^6^-benzoyl­adenine have also been reported: *N* ^6^-benzoyl­adenine–3-hy­droxy­pyridinium-2-carboxyl­ate (1:1) and *N* ^6^-benzoyl­adenine–dl-tartaric acid (1:1) (Karthikeyan *et al.*, 2015 ▸), *N* ^6^-benzoyl­adeninium nitrate (1:1) (Karthikeyan *et al.*, 2015 ▸), *N* ^6^-benzoyl­adenine–adipic acid (1:0.5) (Swinton Darious *et al.*, 2016 ▸). Similarly, various co-crystals of HBA have been reported: 2-amino-4,6-di­methyl­pyrimidine–4-hy­droxy­benzoic acid (Balasubramani *et al.*, 2006 ▸), 4-hy­droxy­benzoic acid–1*H*-imidazole (Wang *et al.*, 2009 ▸), 2-amino-5-bromo­pyridine–4-hy­droxy­benzoic acid (Quah *et al.*, 2010 ▸) and 4,6-dimeth­oxy-2-(methyl­sulfan­yl)-pyrimidine–4-hy­droxy­benzoic acid (Thanigaimani *et al.*, 2012 ▸).

## Synthesis and crystallization   

The title co-crystal was prepared by mixing a hot ethanol solution of *N* ^6^-benzoyl­adenine (30 mg) and 4-hy­droxy­benzoic acid (35 mg) in an equimolar ratio in a total volume of 30 mL. The mixture was warmed over a water bath for 30 min, filtered, and left aside for a few days. Colourless plate-shaped crystals were collected from the mother solution following slow cooling at room temperature.

## Refinement details   

Crystal data, data collection and structure refinement details are summarized in Table 3 ▸. Hydrogen atoms were readily located in difference-Fourier maps and were subsequently treated as riding atoms in geometrically idealized positions, with C—H = 0.93, N—H = 0.86 and O—H = 0.82 Å, and with *U*_iso_(H) = *kU*_eq_(C,N,O), where *k* = 1.5 for hy­droxy and 1.2 for all other H atoms.

## Supplementary Material

Crystal structure: contains datablock(s) I. DOI: 10.1107/S2056989017002171/hg5481sup1.cif

Structure factors: contains datablock(s) I. DOI: 10.1107/S2056989017002171/hg5481Isup2.hkl

Supporting information file. DOI: 10.1107/S2056989017002171/hg5481Isup3.cml

CCDC reference: 1531929

Additional supporting information:  crystallographic information; 3D view; checkCIF report

## Figures and Tables

**Table 1:** Hydrogen-bond geometry (Å, °) *Cg*3 is the centroid of the C11–C16 phenyl ring.

*D*—H⋯*A*	*D*—H	H⋯*A*	*D*⋯*A*	*D*—H⋯*A*
O2—H2*A*⋯N1	0.82	1.92	2.737 (2)	172
O4—H4⋯N9^i^	0.82	1.98	2.784 (2)	168
N6—H6⋯O3	0.86	1.94	2.778 (2)	166
N7—H7⋯O1	0.86	2.14	2.726 (2)	126
N7—H7⋯O1^ii^	0.86	2.36	3.164 (2)	155
C8—H8⋯*Cg*3^ii^	0.93	2.77	3.646 (2)	157

Symmetry codes: (i) 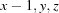; (ii) 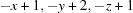.

**Table 2:** Comparison of dihedral angles and torsion angles (°) for various *N* ^6^-benzoyl­adenine-containing crystal structures Pyrimidine ring: N1/C2/N3/C4–C6; imidazole ring of adenine: C4/C5/N7/C8/N9; purine ring system: N1/C2/N3/C4–C6/N7/C8/N9; benzene ring: C11–C16; amide: N6/H6/C10/O1.

Compound	pyrimidine/imidazole	purine/benzene	purine/amide	benzene/amide	C6—N6—C10—C11
*N* ^6^-benzoyl­adenine–DL-tartaric acid^*a*^	2.26 (10)	9.77 (8)	2.93 (18)	11.35 (9)	−179.08 (17)
*N* ^6^-benzoyl­adenine–3-hy­droxy­pridinium-2-carboxyl­ate^*a*^	3.00 (9)	0.94 (8)	21.20 (17)	21.45 (18)	−176.24 (16)
*N* ^6^-benzoyl­adeninium nitrate^*b*^	1.34 (14)	52.25 (12)	23.7 (2)	29.2 (2)	−168.8 (2)
*N* ^6^-benzoyl­adenine–adipic acid^*c*^	0.33 (8)	26.71 (7)	10.8 (7)	23.0 (7)	173.08 (14)
*N* ^6^-benzoyl­adenine–4-hy­droxy­benzoic acid^*d*^	0.24 (12)	70.80 (11)	11.71 (19)	59.4 (2)	−177.91 (18)

References: (*a*) Karthikeyan *et al.* (2015 ▸); (*b*) Karthikeyan *et al.* (2016 ▸); (*c*) Swinton Darious *et al.* (2016 ▸); (*d*) this study.

**Table 3:** Experimental details

Crystal data	
Chemical formula	C_12_H_9_N_5_O·C_7_H_6_O_3_
*M*_r_	377.36
Crystal system, space group	Monoclinic, *P*2_1_/*n*
Temperature (K)	293
*a*, *b*, *c* (Å)	14.7579 (5), 6.7930 (3), 17.2873 (5)
β (°)	91.287 (3)
*V* (Å^3^)	1732.62 (11)
*Z*	4
Radiation type	Cu *K*α
μ (mm^−1^)	0.88
Crystal size (mm)	0.20 × 0.15 × 0.03

Data collection	
Diffractometer	Agilent SuperNova, Dual, Cu at zero, Atlas
Absorption correction	Multi-scan (*CrysAlis PRO*; Agilent, 2013 ▸)
*T*_min_, *T*_max_	0.597, 1.000
No. of measured, independent and observed [*I* > 2σ(*I*)] reflections	6790, 3284, 2457
*R*_int_	0.028
(sin θ/λ)_max_ (Å^−1^)	0.610

Refinement	
*R*[*F* ^2^ > 2σ(*F* ^2^)], *wR*(*F* ^2^), *S*	0.053, 0.161, 1.02
No. of reflections	3284
No. of parameters	256
H-atom treatment	H-atom parameters constrained
Δρ_max_, Δρ_min_ (e Å^−3^)	0.44, −0.30

Computer programs: *CrysAlis PRO* (Agilent, 2013 ▸), *SUPERFLIP* (Palatinus & Chapuis, 2007 ▸), *SHELXL2014* (Sheldrick, 2015 ▸), *PLATON* (Spek, 2009 ▸) and *Mercury* (Macrae *et al.*, 2008 ▸).

## supplementary crystallographic information

### Crystal data

**Table:**

C_12_H_9_N_5_O·C_7_H_6_O_3_	*F*(000) = 784
*M**_r_* = 377.36	*D*_x_ = 1.447 Mg m^−^^3^
Monoclinic, *P*2_1_/*n*	Cu *K*α radiation, λ = 1.54184 Å
*a* = 14.7579 (5) Å	Cell parameters from 2120 reflections
*b* = 6.7930 (3) Å	θ = 3.9–74.6°
*c* = 17.2873 (5) Å	µ = 0.88 mm^−^^1^
β = 91.287 (3)°	*T* = 293 K
*V* = 1732.62 (11) Å^3^	Plate, colorless
*Z* = 4	0.20 × 0.15 × 0.03 mm

### Data collection

**Table:**

Agilent SuperNova, Dual, Cu at zero, Atlas diffractometer	3284 independent reflections
Radiation source: SuperNova (Cu) X-ray Source	2457 reflections with *I* > 2σ(*I*)
Detector resolution: 10.4933 pixels mm^-1^	*R*_int_ = 0.028
ω scans	θ_max_ = 70.1°, θ_min_ = 3.9°
Absorption correction: multi-scan (CrysAlis PRO; Agilent, 2013)	*h* = −12→17
*T*_min_ = 0.597, *T*_max_ = 1.000	*k* = −8→7
6790 measured reflections	*l* = −19→21

### Refinement

**Table:**

Refinement on *F*^2^	Hydrogen site location: inferred from neighbouring sites
Least-squares matrix: full	H-atom parameters constrained
*R*[*F*^2^ > 2σ(*F*^2^)] = 0.053	*w* = 1/[σ^2^(*F*_o_^2^) + (0.0934*P*)^2^ + 0.2078*P*] where *P* = (*F*_o_^2^ + 2*F*_c_^2^)/3
*wR*(*F*^2^) = 0.161	(Δ/σ)_max_ < 0.001
*S* = 1.02	Δρ_max_ = 0.44 e Å^−^^3^
3284 reflections	Δρ_min_ = −0.30 e Å^−^^3^
256 parameters	Extinction correction: SHELXL2014 (Sheldrick, 2015), Fc^*^=kFc[1+0.001xFc^2^λ^3^/sin(2θ)]^-1/4^
0 restraints	Extinction coefficient: 0.0007 (2)

### Special details

**Table:**

Geometry. All esds (except the esd in the dihedral angle between two l.s. planes) are estimated using the full covariance matrix. The cell esds are taken into account individually in the estimation of esds in distances, angles and torsion angles; correlations between esds in cell parameters are only used when they are defined by crystal symmetry. An approximate (isotropic) treatment of cell esds is used for estimating esds involving l.s. planes.

### Fractional atomic coordinates and isotropic or equivalent isotropic displacement parameters (Å^2^)

**Table:**

	*x*	*y*	*z*	*U*_iso_*/*U*_eq_	
O1	0.42682 (11)	1.0650 (3)	0.54392 (9)	0.0705 (6)	
N1	0.39444 (11)	0.9505 (3)	0.78054 (9)	0.0441 (4)	
N3	0.54449 (12)	0.9582 (3)	0.83693 (10)	0.0507 (5)	
N6	0.35504 (10)	0.9706 (2)	0.65248 (9)	0.0398 (4)	
H6	0.3024	0.9381	0.6686	0.048*	
N7	0.57481 (11)	0.9980 (3)	0.63742 (10)	0.0428 (4)	
H7	0.5598	1.0053	0.5892	0.051*	
N9	0.66312 (11)	0.9887 (3)	0.74394 (11)	0.0466 (4)	
C2	0.45586 (14)	0.9466 (4)	0.84004 (12)	0.0522 (6)	
H2	0.4322	0.9340	0.8892	0.063*	
C4	0.57400 (13)	0.9747 (3)	0.76459 (12)	0.0402 (4)	
C6	0.42445 (13)	0.9683 (3)	0.70853 (11)	0.0362 (4)	
C5	0.51711 (12)	0.9806 (3)	0.69794 (11)	0.0357 (4)	
C8	0.65938 (13)	1.0017 (3)	0.66780 (13)	0.0473 (5)	
H8	0.7107	1.0125	0.6378	0.057*	
C10	0.35828 (13)	1.0163 (3)	0.57653 (12)	0.0430 (5)	
C11	0.26848 (13)	1.0088 (3)	0.53510 (11)	0.0448 (5)	
C12	0.21996 (19)	0.8373 (5)	0.53086 (16)	0.0774 (8)	
H12	0.2415	0.7252	0.5561	0.093*	
C13	0.1393 (2)	0.8303 (7)	0.4892 (2)	0.1083 (14)	
H13	0.1075	0.7124	0.4847	0.130*	
C14	0.1062 (2)	0.9956 (7)	0.45466 (18)	0.0950 (13)	
H14	0.0510	0.9908	0.4277	0.114*	
C15	0.15326 (18)	1.1700 (6)	0.45909 (15)	0.0828 (10)	
H15	0.1297	1.2828	0.4356	0.099*	
C16	0.23613 (16)	1.1771 (4)	0.49876 (14)	0.0621 (6)	
H16	0.2694	1.2933	0.5009	0.075*	
O2	0.22103 (10)	0.9691 (3)	0.83296 (9)	0.0563 (4)	
H2A	0.2718	0.9530	0.8157	0.084*	
O3	0.18126 (10)	0.9349 (4)	0.70936 (9)	0.0734 (6)	
O4	−0.20332 (10)	1.0061 (3)	0.86009 (10)	0.0608 (5)	
H4	−0.2373	0.9926	0.8223	0.091*	
C17	0.15996 (13)	0.9583 (3)	0.77584 (11)	0.0409 (4)	
C18	0.06559 (12)	0.9737 (3)	0.79947 (11)	0.0366 (4)	
C19	0.04097 (14)	1.0009 (3)	0.87579 (11)	0.0435 (5)	
H19	0.0857	1.0112	0.9143	0.052*	
C20	−0.04879 (14)	1.0128 (4)	0.89506 (12)	0.0499 (5)	
H20	−0.0643	1.0314	0.9464	0.060*	
C21	−0.11692 (13)	0.9970 (3)	0.83780 (12)	0.0425 (5)	
C22	−0.09232 (13)	0.9722 (3)	0.76111 (12)	0.0437 (5)	
H22	−0.1368	0.9637	0.7223	0.052*	
C23	−0.00281 (13)	0.9602 (3)	0.74278 (12)	0.0433 (5)	
H23	0.0127	0.9427	0.6914	0.052*	

### Atomic displacement parameters (Å^2^)

**Table:**

	*U*^11^	*U*^22^	*U*^33^	*U*^12^	*U*^13^	*U*^23^
O1	0.0375 (9)	0.1294 (16)	0.0446 (8)	−0.0059 (9)	−0.0021 (6)	0.0179 (9)
N1	0.0314 (8)	0.0663 (11)	0.0343 (8)	0.0027 (7)	−0.0060 (6)	−0.0018 (7)
N3	0.0361 (9)	0.0755 (12)	0.0400 (9)	0.0026 (8)	−0.0107 (7)	−0.0030 (8)
N6	0.0240 (8)	0.0597 (10)	0.0353 (8)	−0.0021 (6)	−0.0076 (6)	0.0019 (7)
N7	0.0287 (8)	0.0592 (10)	0.0403 (9)	−0.0002 (7)	−0.0034 (6)	0.0011 (7)
N9	0.0264 (8)	0.0624 (11)	0.0503 (10)	0.0015 (7)	−0.0087 (7)	−0.0032 (8)
C2	0.0379 (11)	0.0841 (16)	0.0344 (9)	0.0035 (10)	−0.0064 (8)	−0.0016 (10)
C4	0.0302 (10)	0.0476 (10)	0.0424 (10)	0.0019 (7)	−0.0097 (7)	−0.0034 (8)
C6	0.0296 (9)	0.0430 (10)	0.0355 (9)	0.0010 (7)	−0.0078 (7)	−0.0021 (7)
C5	0.0302 (9)	0.0401 (9)	0.0365 (9)	0.0011 (7)	−0.0061 (7)	−0.0012 (7)
C8	0.0259 (10)	0.0649 (13)	0.0511 (12)	0.0005 (8)	−0.0012 (8)	0.0007 (10)
C10	0.0301 (10)	0.0615 (12)	0.0372 (10)	0.0017 (8)	−0.0048 (7)	0.0011 (9)
C11	0.0311 (10)	0.0710 (13)	0.0320 (9)	0.0012 (9)	−0.0061 (7)	0.0023 (9)
C12	0.0706 (17)	0.0902 (19)	0.0699 (16)	−0.0204 (15)	−0.0324 (13)	0.0151 (15)
C13	0.086 (2)	0.150 (3)	0.086 (2)	−0.053 (2)	−0.0499 (18)	0.030 (2)
C14	0.0481 (15)	0.182 (4)	0.0536 (15)	−0.0141 (19)	−0.0213 (12)	0.0164 (19)
C15	0.0563 (15)	0.135 (3)	0.0564 (14)	0.0353 (18)	−0.0098 (11)	0.0172 (17)
C16	0.0517 (13)	0.0784 (16)	0.0558 (12)	0.0135 (12)	−0.0075 (10)	0.0073 (12)
O2	0.0294 (7)	0.0975 (12)	0.0416 (8)	−0.0001 (7)	−0.0052 (6)	−0.0040 (8)
O3	0.0339 (8)	0.1439 (18)	0.0425 (8)	−0.0016 (9)	−0.0009 (6)	−0.0160 (10)
O4	0.0290 (8)	0.1037 (14)	0.0497 (9)	0.0011 (7)	−0.0003 (6)	−0.0057 (9)
C17	0.0317 (10)	0.0510 (11)	0.0398 (10)	−0.0015 (8)	−0.0052 (7)	−0.0014 (8)
C18	0.0308 (10)	0.0403 (9)	0.0383 (9)	−0.0009 (7)	−0.0051 (7)	0.0018 (7)
C19	0.0332 (10)	0.0621 (12)	0.0350 (9)	−0.0004 (8)	−0.0078 (7)	0.0008 (8)
C20	0.0349 (10)	0.0822 (15)	0.0324 (9)	0.0006 (10)	−0.0023 (8)	0.0000 (10)
C21	0.0294 (10)	0.0540 (11)	0.0439 (10)	−0.0002 (8)	−0.0034 (8)	0.0009 (9)
C22	0.0330 (10)	0.0578 (12)	0.0397 (10)	0.0009 (8)	−0.0092 (7)	−0.0036 (9)
C23	0.0346 (10)	0.0602 (12)	0.0349 (9)	0.0014 (8)	−0.0060 (7)	−0.0029 (9)

### Geometric parameters (Å, º)

**Table:**

O1—C10	1.215 (3)	C13—H13	0.9300
N1—C6	1.336 (3)	C14—C15	1.375 (5)
N1—C2	1.356 (2)	C14—H14	0.9300
N3—C2	1.313 (3)	C15—C16	1.389 (3)
N3—C4	1.338 (3)	C15—H15	0.9300
N6—C10	1.351 (3)	C16—H16	0.9300
N6—C6	1.394 (2)	O2—C17	1.324 (2)
N6—H6	0.8600	O2—H2A	0.8200
N7—C8	1.343 (2)	O3—C17	1.209 (3)
N7—C5	1.369 (3)	O4—C21	1.342 (3)
N7—H7	0.8600	O4—H4	0.8200
N9—C8	1.319 (3)	C17—C18	1.464 (3)
N9—C4	1.374 (3)	C18—C19	1.389 (3)
C2—H2	0.9300	C18—C23	1.394 (2)
C4—C5	1.411 (2)	C19—C20	1.376 (3)
C6—C5	1.386 (3)	C19—H19	0.9300
C8—H8	0.9300	C20—C21	1.399 (3)
C10—C11	1.493 (3)	C20—H20	0.9300
C11—C12	1.369 (4)	C21—C22	1.393 (3)
C11—C16	1.384 (3)	C22—C23	1.368 (3)
C12—C13	1.379 (3)	C22—H22	0.9300
C12—H12	0.9300	C23—H23	0.9300
C13—C14	1.357 (5)		
			
C6—N1—C2	118.60 (18)	C14—C13—H13	120.0
C2—N3—C4	112.88 (17)	C12—C13—H13	120.0
C10—N6—C6	129.53 (17)	C13—C14—C15	120.8 (3)
C10—N6—H6	115.2	C13—C14—H14	119.6
C6—N6—H6	115.2	C15—C14—H14	119.6
C8—N7—C5	106.93 (17)	C14—C15—C16	119.7 (3)
C8—N7—H7	126.5	C14—C15—H15	120.2
C5—N7—H7	126.5	C16—C15—H15	120.2
C8—N9—C4	104.19 (16)	C11—C16—C15	119.1 (3)
N3—C2—N1	128.1 (2)	C11—C16—H16	120.4
N3—C2—H2	115.9	C15—C16—H16	120.4
N1—C2—H2	115.9	C17—O2—H2A	109.5
N3—C4—N9	125.60 (17)	C21—O4—H4	109.5
N3—C4—C5	124.43 (18)	O3—C17—O2	121.96 (18)
N9—C4—C5	109.97 (18)	O3—C17—C18	122.96 (17)
N1—C6—C5	118.50 (16)	O2—C17—C18	115.08 (18)
N1—C6—N6	113.25 (17)	C19—C18—C23	118.42 (18)
C5—C6—N6	128.25 (18)	C19—C18—C17	123.06 (17)
N7—C5—C6	137.61 (17)	C23—C18—C17	118.53 (18)
N7—C5—C4	104.93 (16)	C20—C19—C18	120.79 (18)
C6—C5—C4	117.46 (18)	C20—C19—H19	119.6
N9—C8—N7	113.98 (18)	C18—C19—H19	119.6
N9—C8—H8	123.0	C19—C20—C21	120.3 (2)
N7—C8—H8	123.0	C19—C20—H20	119.8
O1—C10—N6	124.15 (18)	C21—C20—H20	119.8
O1—C10—C11	121.74 (18)	O4—C21—C22	123.28 (18)
N6—C10—C11	114.07 (17)	O4—C21—C20	117.8 (2)
C12—C11—C16	120.3 (2)	C22—C21—C20	118.97 (19)
C12—C11—C10	120.8 (2)	C23—C22—C21	120.10 (17)
C16—C11—C10	118.9 (2)	C23—C22—H22	119.9
C11—C12—C13	120.1 (3)	C21—C22—H22	119.9
C11—C12—H12	119.9	C22—C23—C18	121.40 (19)
C13—C12—H12	119.9	C22—C23—H23	119.3
C14—C13—C12	119.9 (3)	C18—C23—H23	119.3
			
C4—N3—C2—N1	−0.4 (4)	N6—C10—C11—C12	−60.8 (3)
C6—N1—C2—N3	0.0 (4)	O1—C10—C11—C16	−56.9 (3)
C2—N3—C4—N9	−179.7 (2)	N6—C10—C11—C16	121.2 (2)
C2—N3—C4—C5	0.4 (3)	C16—C11—C12—C13	1.1 (5)
C8—N9—C4—N3	179.9 (2)	C10—C11—C12—C13	−176.8 (3)
C8—N9—C4—C5	−0.2 (2)	C11—C12—C13—C14	−2.4 (6)
C2—N1—C6—C5	0.4 (3)	C12—C13—C14—C15	1.6 (6)
C2—N1—C6—N6	−179.98 (19)	C13—C14—C15—C16	0.6 (5)
C10—N6—C6—N1	168.6 (2)	C12—C11—C16—C15	1.0 (4)
C10—N6—C6—C5	−11.9 (3)	C10—C11—C16—C15	179.0 (2)
C8—N7—C5—C6	−179.9 (2)	C14—C15—C16—C11	−1.9 (4)
C8—N7—C5—C4	0.0 (2)	O3—C17—C18—C19	−179.9 (2)
N1—C6—C5—N7	179.6 (2)	O2—C17—C18—C19	1.0 (3)
N6—C6—C5—N7	0.0 (4)	O3—C17—C18—C23	0.2 (3)
N1—C6—C5—C4	−0.4 (3)	O2—C17—C18—C23	−178.92 (19)
N6—C6—C5—C4	−179.93 (18)	C23—C18—C19—C20	0.5 (3)
N3—C4—C5—N7	179.99 (19)	C17—C18—C19—C20	−179.43 (19)
N9—C4—C5—N7	0.1 (2)	C18—C19—C20—C21	0.2 (3)
N3—C4—C5—C6	0.0 (3)	C19—C20—C21—O4	178.8 (2)
N9—C4—C5—C6	−179.93 (16)	C19—C20—C21—C22	−1.0 (3)
C4—N9—C8—N7	0.2 (2)	O4—C21—C22—C23	−178.7 (2)
C5—N7—C8—N9	−0.2 (2)	C20—C21—C22—C23	1.1 (3)
C6—N6—C10—O1	0.1 (4)	C21—C22—C23—C18	−0.4 (3)
C6—N6—C10—C11	−177.92 (19)	C19—C18—C23—C22	−0.3 (3)
O1—C10—C11—C12	121.1 (3)	C17—C18—C23—C22	179.55 (19)

### Hydrogen-bond geometry (Å, º)

Cg3 is the centroid of the C11–C16 phenyl ring.

**Table:**

*D*—H···*A*	*D*—H	H···*A*	*D*···*A*	*D*—H···*A*
O2—H2*A*···N1	0.82	1.92	2.737 (2)	172
O4—H4···N9^i^	0.82	1.98	2.784 (2)	168
N6—H6···O3	0.86	1.94	2.778 (2)	166
N7—H7···O1	0.86	2.14	2.726 (2)	126
N7—H7···O1^ii^	0.86	2.36	3.164 (2)	155
C8—H8···*Cg*3^ii^	0.93	2.77	3.646 (2)	157

Symmetry codes: (i) *x*−1, *y*, *z*; (ii) −*x*+1, −*y*+2, −*z*+1.
